# Structure and *in vivo* psoralen DNA crosslink repair activity of mycobacterial Nei2

**DOI:** 10.1128/mbio.01248-24

**Published:** 2024-07-16

**Authors:** Garrett M. Warren, Stewart Shuman

**Affiliations:** 1 Molecular Biology Program, Memorial Sloan Kettering Cancer Center, New York, New York, USA; New York University School of Medicine, New York, New York, USA

**Keywords:** DNA repair, inter-strand crosslinks, AP lyase, DNA glycosylase, DNA helicase

## Abstract

*Mycobacterium smegmatis* Nei2 is a monomeric enzyme with AP β-lyase activity on single-stranded DNA. Expression of Nei2, and its operonic neighbor Lhr (a tetrameric 3′-to-5′ helicase), is induced in mycobacteria exposed to DNA damaging agents. Here, we find that *nei2* deletion sensitizes *M. smegmatis* to killing by DNA inter-strand crosslinker trimethylpsoralen but not to crosslinkers mitomycin C and cisplatin. By contrast, deletion of *lhr* sensitizes to killing by all three crosslinking agents. We report a 1.45 Å crystal structure of recombinant Nei2, which is composed of N and C terminal lobes flanking a central groove suitable for DNA binding. The C lobe includes a tetracysteine zinc complex. Mutational analysis identifies the N-terminal proline residue (Pro2 of the ORF) and Lys51, but not Glu3, as essential for AP lyase activity. We find that Nei2 has 5-hydroxyuracil glycosylase activity on single-stranded DNA that is effaced by alanine mutations of Glu3 and Lys51 but not Pro2. Testing complementation of psoralen sensitivity by expression of wild-type and mutant *nei2* alleles in ∆*nei2* cells established that AP lyase activity is neither sufficient nor essential for crosslink repair. By contrast, complementation of psoralen sensitivity of ∆*lhr* cells by mutant *lhr* alleles depended on Lhr’s ATPase/helicase activities and its tetrameric quaternary structure. The *lhr–nei2* operon comprises a unique bacterial system to rectify inter-strand crosslinks.

**IMPORTANCE:**

The DNA inter-strand crosslinking agents mitomycin C, cisplatin, and psoralen–UVA are used clinically for the treatment of cancers and skin diseases; they have been invaluable in elucidating the pathways of inter-strand crosslink repair in eukaryal systems. Whereas DNA crosslinkers are known to trigger a DNA damage response in bacteria, the roster of bacterial crosslink repair factors is incomplete and likely to vary among taxa. This study implicates the DNA damage-inducible mycobacterial *lhr–nei2* gene operon in protecting *Mycobacterium smegmatis* from killing by inter-strand crosslinkers. Whereas interdicting the activity of the Lhr helicase sensitizes mycobacteria to mitomycin C, cisplatin, and psoralen–UVA, the Nei2 glycosylase functions uniquely in evasion of damage caused by psoralen–UVA.

## INTRODUCTION

**Fig 1:**
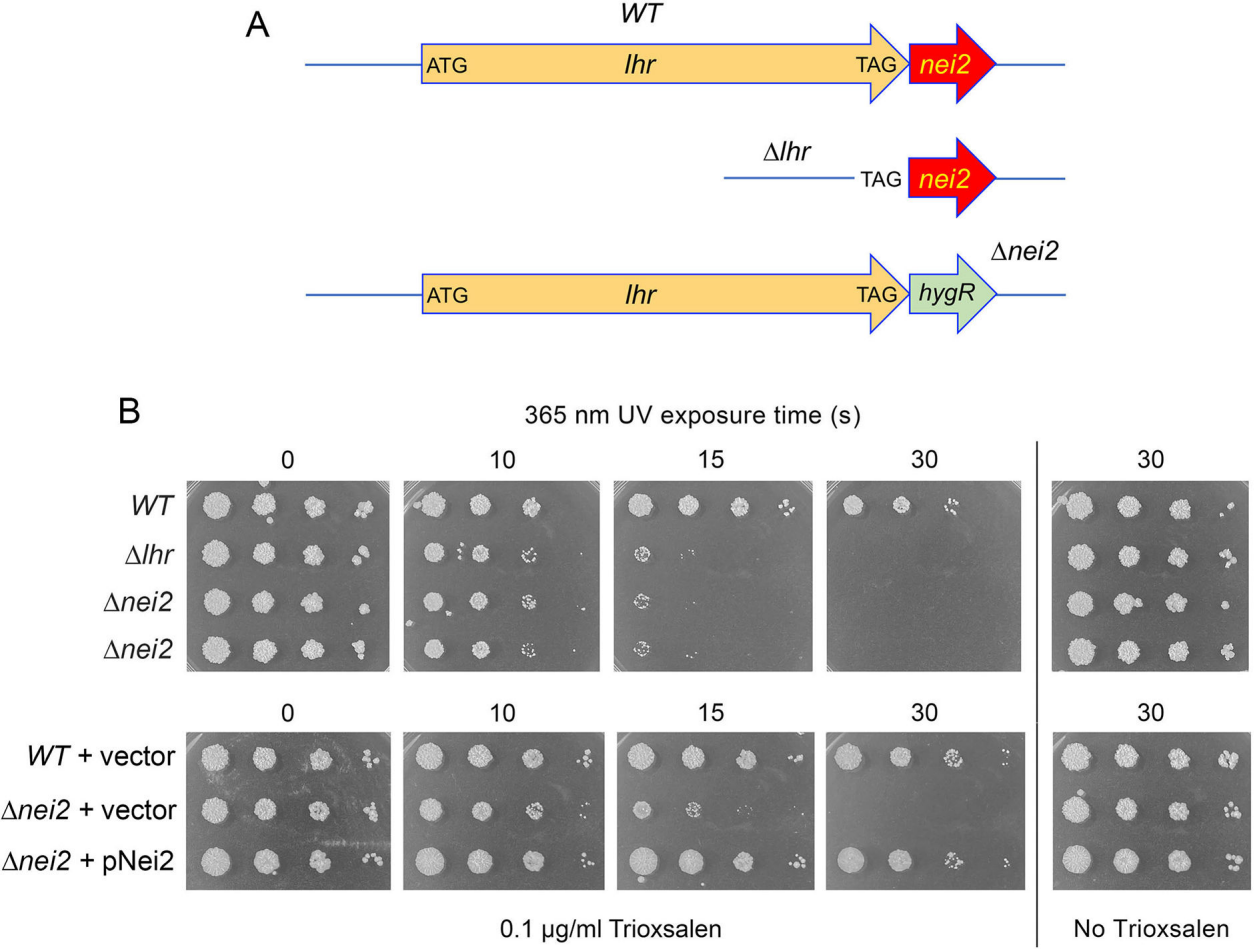
Deletion of *lhr* or *nei2* sensitizes *M. smegmatis* to killing by trioxsalen-UVA. (**A**) Cartoon depiction of the wild-type *M. smegmatis lhr-nei2* operon with the respective open reading frames (ORFs) rendered as arrows. ATG and TAG are the start and stop codons that demarcate the *lhr* ORF. In the genomic locus of the ∆*lhr* strain, the entire *lhr* ORF is deleted and only the *lhr* stop codon remains flanking the unperturbed *nei2* ORF. In the *nei2*∆ strain, the entire *nei2* ORF is deleted and replaced with a hygromycin resistance gene (*hygR*) without perturbing the *lhr* ORF. (**B**) Serial 10-fold dilutions of wild-type, ∆*lhr*, and ∆*nei2* cells (two independent isolates) (*top panel*) or wild-type and ∆*nei2* cells transformed with plasmids pMV261 (empty vector) or pNei2 (*bottom panel*) were spotted on 7H10 agar plates lacking or containing 0.1 µg/mL trioxsalen as specified and exposed to 365 nm light for 0, 10, 15, or 30 s as described under Materials and Methods. The plates were photographed after incubation for 3 days at 37°C.

The *lhr–nei2* gene operon of *Mycobacterium tuberculosis* and its avirulent relative *Mycobacterium smegmatis* encodes a pair of enzymes implicated in DNA repair: the Lhr helicase and the Nei2 AP lyase (1–4) (Fig. 1A). *M. smegmatis* Lhr is a 1507-amino acid (aa) nucleic acid-dependent ATPase/dATPase that uses ATP hydrolysis to drive unidirectional 3′ to 5′ translocation along single-strand DNA and to unwind DNA:DNA or RNA:DNA duplexes *en route* (1). The ATPase, DNA translocase, and helicase activities of Lhr are encompassed within the N-terminal 856-aa segment, referred to as Lhr-Core. The large and structurally distinctive C-terminal domain (CTD) of Lhr (aa 857–1,507), though not required for Lhr’s motor activity, serves to nucleate a homo-tetrameric quaternary structure of full-length Lhr (2, 3). *M. smegmatis* Nei2, so named by virtue of its homology to *Escherichia coli* endonuclease VIII (Nei), is a 252-aa monomeric enzyme with AP β-lyase activity on single-stranded DNA (4). The properties of Nei2 as an AP lyase are consistent with the established mechanism of Nei/Fpg-type glycosylase/lyases, which exploit the secondary amino group of an N-terminal proline residue (corresponding to Pro2 of the *nei*-encoded polypeptide) as a nucleophile that attacks the deoxyribose C1′ of the target nucleoside, leading to expulsion of the nucleobase and formation of a covalent enzyme–DNA intermediate, followed by either one β-elimination step (the predominant outcome of Nei2 action), or a β-elimination step followed by a δ-elimination step, to incise the DNA backbone (5, 6). The finding that *M. smegmatis* Nei2 displays an extremely weak uracil glycosylase activity compared to its more vigorous AP lyase activity, suggested that, if Nei2 does have an intrinsic glycosylase activity, it is targeted to specific DNA lesions other than dU (4).

Although neither *lhr* nor *nei2* is essential for mycobacterial viability under laboratory growth conditions (4, 7, 8), a potential role for the Lhr and Nei2 proteins in mycobacterial DNA repair *in vivo* was suggested by multiple independent reports that the *lhr–nei2* operon is transcriptionally upregulated by the LexA/RecA-independent PafBC pathway in response to DNA damage inflicted by mitomycin C (MMC), or shortwave UV irradiation (UVC), and by treatment with ciprofloxacin (a type II topoisomerase poison) or nargenicin (an inhibitor of the replicative DNA polymerase DnaE1) (9–14).

We recently initiated a genetic analysis of *lhr–nei2* operon function by constructing a ∆*lhr* strain in which the chromosomal *lhr* ORF was deleted cleanly without perturbing the flanking *nei2* gene (Fig. 1A). We found that loss of Lhr sensitized *M. smegmatis* to killing by DNA inter-strand purine crosslinkers MMC and cisplatin but did not sensitize to killing by the monoadduct-forming alkylating agent methyl methanesulfonate (MMS) or by UVC irradiation (4). Testing for complementation of MMC and cisplatin sensitivity by expression of Lhr mutants in ∆*lhr* cells showed that Lhr’s ATPase motor and its tetrameric quaternary structure were essential for Lhr function in crosslink repair *in vivo* (4).

In the present study, we conducted a genetic and structural analysis of *M. smegmatis* Nei2. We find that deleting the *nei2* ORF without perturbing the upstream *lhr* gene (Fig. 1A) did not recapitulate the MMC and cisplatin sensitivity seen in the ∆*lhr* mutant, nor were ∆*nei2* cells sensitized to killing by MMS and UVC. Rather, ∆*nei2* cells (as well as ∆*lhr* cells) were sensitized to killing by a different photoactivated crosslinking agent, psoralen–UVA, which produces inter-strand pyrimidine crosslinks. Guided by a crystal structure of *M. smegmatis* Nei2, we interrogated the effect of active site mutations on AP lyase activity *in vitro*. By testing our mutants for complementation of ∆*nei2*, we determined that Nei2 AP lyase activity is neither sufficient nor necessary for Nei2 function in evading death by psoralen–UVA. We demonstrate here that Nei2 has 5-hydroxyuracil glycosylase/β-lyase activity on single-stranded DNA. Our results instate the tetrameric Lhr helicase as the linchpin of a novel bacterial DNA repair pathway, effective against chemically diverse inter-strand crosslinks, in which the Nei2 protein functions specifically in the context of psoralen–UVA-induced lesions.

## RESULTS

### Deletion of *lhr* or *nei2* sensitizes *M. smegmatis* to killing by trioxsalen–UVA

Having shown previously that deletion of the *lhr* gene of the *lhr–nei2* operon confers extreme sensitivity to inter-strand purine nucleobase crosslinkers MMC and cisplatin (4), it was of interest to interrogate the effect of a different DNA crosslinking agent, trimethylpsoralen (trioxsalen). Trioxsalen intercalates into DNA and forms single-strand adducts and inter-strand crosslinks after photoactivation with 365 nm UV light (UVA), via cycloaddition to the 5,6 double bond of pyrimidines, preferentially at the thymine bases of 5′-TA dinucleotides (15). For this purpose, we deployed the ∆*lhr* strain generated previously and a newly constructed ∆*nei2* mutant in which the *nei2* ORF was replaced by a *hygR* cassette without perturbing the upstream *lhr* gene (Fig. 1A). Serial dilutions of logarithmically growing wild-type, ∆*lhr*, and ∆*nei2* cells were spotted on agar plates containing 0.1 µg/mL trioxsalen, incubated for 2 h to allow uptake of the compound, and then exposed to 365 nm UV light from a fixed distance above the plate for 10, 15, or 30 s. The plates were then incubated for 3 days to gauge survival. Control tests showed that: (i) 30 s of 365 nm UV exposure had no effect on cell growth in the absence of trioxsalen; and (ii) 0.1 µg/mL trioxsalen had no impact on cell growth in the absence of UVA irradiation (Fig. 1B). The salient findings were that ∆*lhr* and ∆*nei2* cells were equally and exquisitely sensitive to killing by trioxsalen compared to wild type, by approximately 100-fold after 15 s of UVA exposure and 1,000-fold after 30 s of exposure (Fig. 1B).

To exclude the possibility that the psoralen sensitivity of ∆*nei2* cells was caused by an unintended effect on the expression of the upstream *lhr* gene, we performed two control experiments. First, we assayed the presence of the Lhr protein in whole cell extracts of wild type, ∆*lhr*, and ∆*nei2* cells by Western blotting with anti-Lhr antibody (4), which confirmed that the ~175 kDa Lhr polypeptide was present at similar steady-state levels in wild-type and ∆*nei2* cells but absent in ∆*lhr* cells (Fig. S1). Second, we demonstrated that the introduction of a plasmid pNei2 expressing the *nei2* ORF (under the control of the *lhr–nei2* operon promoter) into ∆*nei2* cells restored resistance to trioxsalen–UVA equivalent to that of wild-type cells (Fig. 1B).

As noted above, psoralen forms both nucleobase monoadducts and inter-strand crosslinks (16), as do MMC and cisplatin. Cisplatin forms intra-strand G-G and G-A crosslinks, inter-strand G-G crosslinks, and G-monoadducts via reaction with purine-N7 in the major groove (17). MMC reacts with guanine bases at 5′-CpG sites to form inter-strand G-G crosslinks and G-monoadducts via reaction with guanine-N2 in the DNA minor groove (18). We reported previously that genetic ablation of the UvrD1 helicase, a key agent of nucleotide excision repair (19), sensitized *M. smegmatis* to killing by MMC and cisplatin and that combining ∆*lhr* and ∆*uvrD1* null alleles exerted an additive effect on sensitivity to these two clastogens (4). Here, we found that ∆*uvrD1* cells are more sensitive to trioxsalen–UVA treatment than ∆*lhr* cells. This is apparent at the earliest UVA exposure time (5 s), where the survival of ∆*uvrD1* cells is diminished by at least 10-fold while wild-type and ∆*lhr* cells are unaffected (Fig. S2). The ∆*uvrD1* ∆*lhr* double-mutant is more sensitive to killing after 10 s of UVA irradiation than the ∆*uvrD1* single-mutant (Fig. S2).

### Deletion of *nei2* does not sensitize *M. smegmatis* to killing by cisplatin and MMC

**Fig 2:**
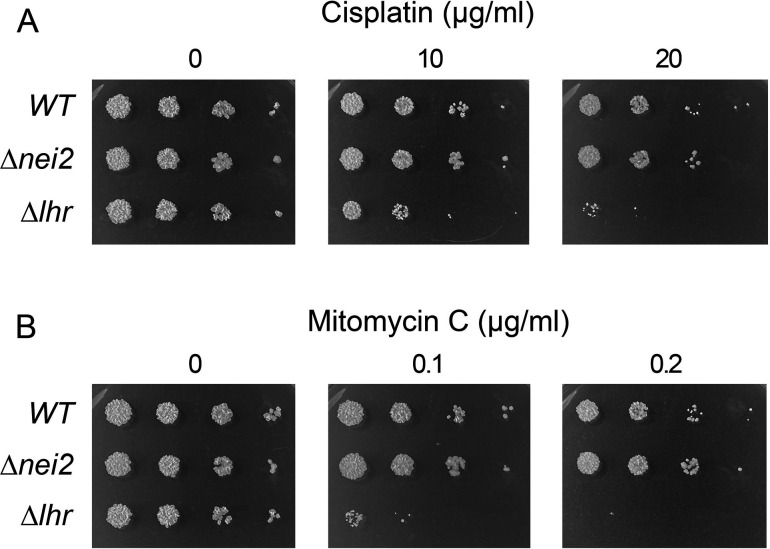
Deletion of *nei2* does not sensitize *M. smegmatis* to killing by MMC and cisplatin. Wild-type, ∆*nei2*, and ∆*lhr* cells were treated with either 0, 10, or 20 µg/mL cisplatin for 1 h at 37°C (panel A) or 0, 0.1, or 0.2 µg/mL MMC for 2 h at 37°C (panel B). Post-treatment, the cells were harvested by centrifugation, washed two times to remove the clastogen, then resuspended and adjusted to equal optical density. Serial 10-fold dilutions were spotted on 7H10 agar plates and incubated for 3 days at 37°C to gauge survival.

We queried the sensitivity of ∆*nei2* cells to killing by cisplatin and MMC in comparison to wild-type *M. smegmatis* and the cisplatin/MMC-sensitive ∆*lhr* strain. Logarithmically growing cultures were mock-treated or exposed to either 10 or 20 µg/mL cisplatin for 1 h (Fig. 2A) or 0.1 or 0.2 µg/mL MMC for 2 h (Fig. 2B). Cells were then harvested, washed to remove residual cisplatin or MMC, and serial 10-fold dilutions were spotted on 7H10 agar plates to gauge survival. Although the ∆*lhr* strain was killed by transient exposure to cisplatin and MMC, the *nei2*∆ strain was no more sensitive than wild-type *M. smegmatis* (Fig. 2). Thus, Nei2 is implicated specifically in the repair of psoralen–UVA crosslinks, while Lhr participates in the repair of three chemically distinct types of DNA crosslinks.

### *nei2* deletion does not sensitize *M. smegmatis* to killing by MMS or UVC irradiation

**Fig 3:**
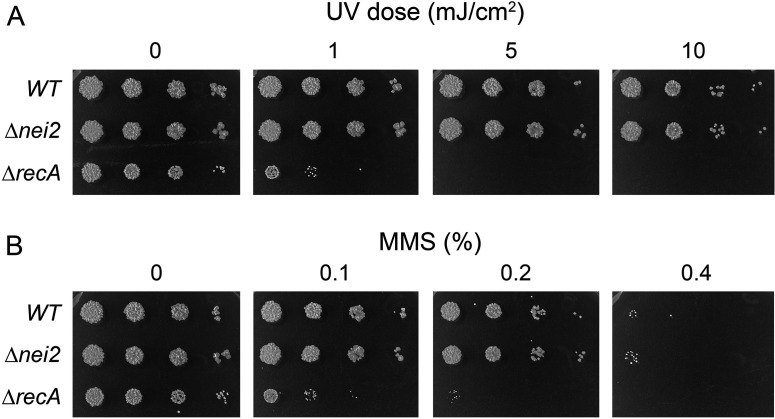
Deletion of *nei2* does not sensitize *M. smegmatis* to killing by MMS or UVC. (**A**) Serial 10-fold dilutions of wild-type, *nei2,* and ∆r*ecA* cells (adjusted for optical density) were spotted on 7H10 agar plates, which were exposed to the UV doses specified (0, 1, 5, or 10 mJ/cm^2^). The plates were photographed after incubation in the dark for 3 days at 37°C. (**B**) Wild-type, ∆*nei2,* and ∆*recA* cells were treated with 0%, 0.1%, 0.2%, or 0.4% MMS for 1 h at 37°C. Post-treatment, sodium thiosulfate was added to 5% final concentration, cells were harvested and washed, and serial 10-fold dilutions of cells were spotted on 7H10 agar plates and incubated for 3 days at 37°C to gauge survival.

Short-wave UVC radiation causes two classes of intra-strand DNA lesions—cyclobutane pyrimidine dimers and 6-4 photoproducts—that are repaired in *M. smegmatis* via NER, as evinced by the exquisite sensitivity of ∆*uvrD1* cells to killing by UVC (19). To query whether the ∆*nei2* mutant is UVC sensitive, we spotted serial dilutions of wild-type and ∆*nei2* cells on 7H10 agar plates, along with ∆*recA* cells (as a positive control for enhanced UVC killing). The plates were then mock-treated or exposed to escalating doses (1, 5, or 10 mJ/cm^2^) of 254 nm UV light. Following treatment, the plates were wrapped in foil (to prevent repair by photolyase) and incubated at 37°C. The wild-type and ∆*nei2* strains were equally resistant to 1 and 5 mJ/cm^2^ and slightly sensitive to 10 mJ/cm^2^, while the ∆*recA* strain suffered a ~100-fold reduction in survival at 1 mJ/cm^2^ and a >1,000-fold reduction in survival at 5 mJ/cm^2^ (Fig. 3A).

MMS is a DNA alkylating agent that generates 7-methyl-G and 3-methyl-A base monoadducts. We found that wild-type and ∆*nei2* cells were equally responsive to 1 h treatments with 0.2% and 0.4% MMS, whereby viability of both strains was reduced sharply by 0.4% MMS (Fig. 3B). As expected, ∆*recA* cells were killed by 0.1% and 0.2% MMS, which had no or little effect on wild-type and ∆*nei2* cells (Fig. 3B).

### Atomic structure of *M. smegmatis* Nei2

**Fig 4:**
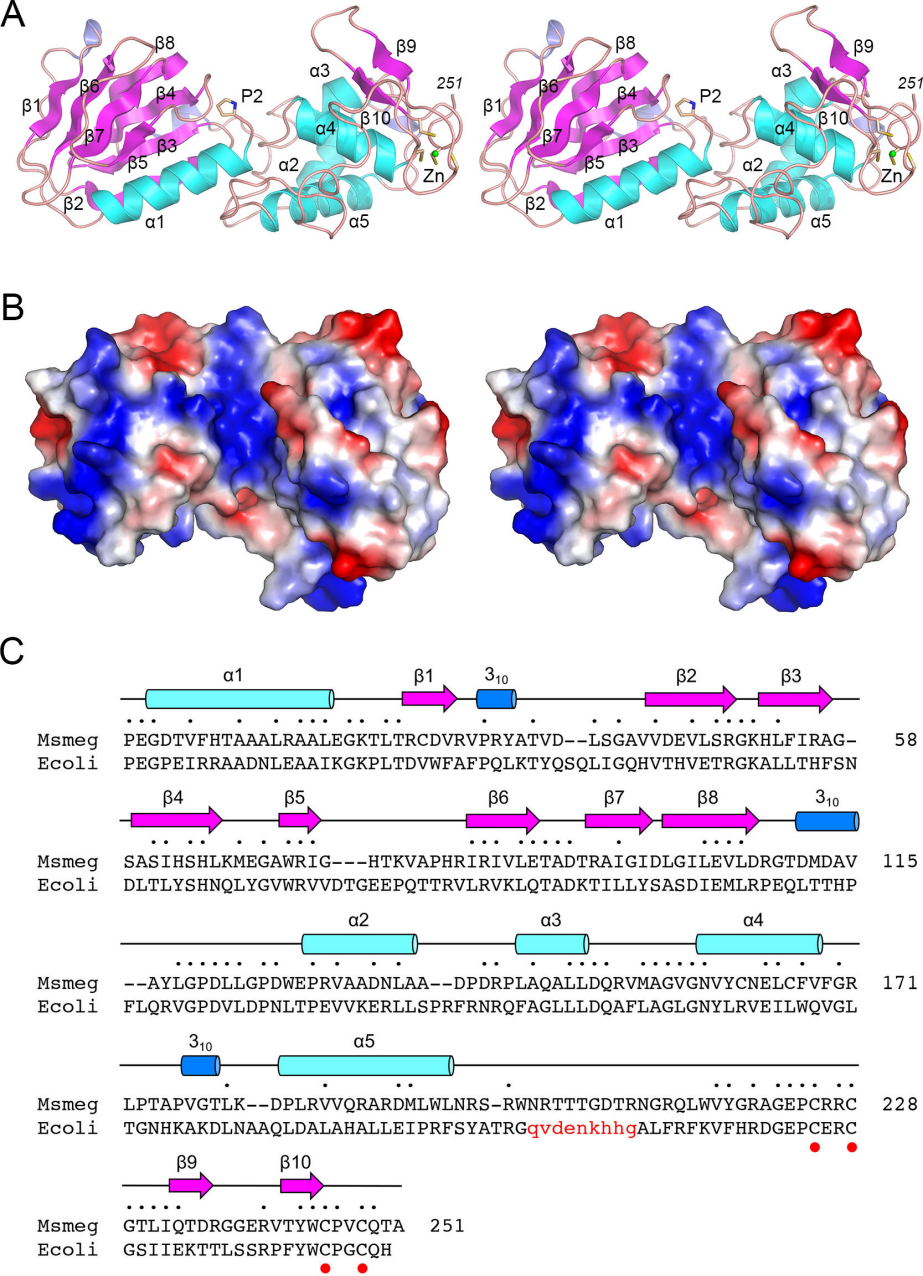
Atomic structure of Nei2. (A) Stereo view of the tertiary structure of Nei2, depicted as a cartoon model with magenta β-strands, cyan α-helices, and blue 3_10_-helices. The secondary structure elements are labeled according to their order in the primary structure, as shown in panel (C). The N-terminal proline (P2) that forms a Schiff base intermediate in the AP lyase reaction is depicted as a stick model. A zinc ion (green sphere) is coordinated by four cysteines near the C-terminus of Nei2. (B) Stereo view of a surface electrostatic model of Nei2, looking down into the electropositive groove between the N and C lobes of the enzyme. (C) Dali alignment of the amino acid sequences of *M. smegmatis* Nei2 and *E. coli* Nei. Positions of side chain identity/similarity are indicated by dots above the alignment. A segment of EcoNei that is disordered in the crystal structure (PDB 1K3X) is depicted in lowercase red font. The secondary structure elements of Nei2 are shown above the primary structure, with β-strands depicted as magenta arrows, α-helices as cyan cylinders, and 3_10_-helices as blue cylinders. The four cysteines that coordinate a zinc ion bond are denoted by red dots below the alignment.

Purified recombinant Nei2 with a C-terminal His_6_-tag is a monomeric enzyme with AP lyase activity on single-stranded DNA (4). Here, we crystalized Nei2 and determined its structure at 1.45 Å resolution. The refined Nei2 model (R_work_/R_free_ = 16.7/20.5) comprised a continuous polypeptide from Pro2 to Ala251. There was no density for the N-terminal methionine, which we presume was removed by methionine aminopeptidase during Nei2 expression in *E. coli*. There was no interpretable density for the C-terminal Ser252 of the native Nei2 polypeptide or the appended C-terminal His_6_-tag. The Nei2 tertiary structure is depicted in stereo in Fig. 4A and consists of 5 α-helices, 10 β-strands, and 3 3_10_-helices, numbered sequentially and displayed over the Nei2 amino acid sequence in Fig. 4C. The protein consists of an N-terminal lobe (aa 2–116) and a C-terminal lobe (aa 123–251) connected by a linker peptide. The N lobe is built around two four-strand antiparallel β-sheets (β1↑•β6↓•β7↑•β5↓ and β2↑•β3↓•β4↑•β8↓) that form a β-sandwich, which is flanked by the N-terminal α1 helix and two short 3_10_ helices (Fig. 4A). The C domain comprises a 4-helix bundle, a β9-β10 hairpin, and a tetracysteine-zinc complex (Fig. 4A). A strong anomalous difference peak was detected overlying the modeled zinc atom (Fig. S3). Weaker anomalous peaks were detected over the Sγ atoms of Cys225, Cys228, Cys245, and Cys248 that bind the zinc ion (Fig. S3). The N and C lobes are arranged in a saddle-like shape with a deep groove between them, in which the N-terminal proline (Pro2) is located, thereby demarcating the AP lyase active site (Fig. 4A and B). The saddle groove is lined by positive electrostatic potential that provides a docking site for the DNA substrate, as seen in structures of AP lyase-related enzymes.

**Fig 5:**
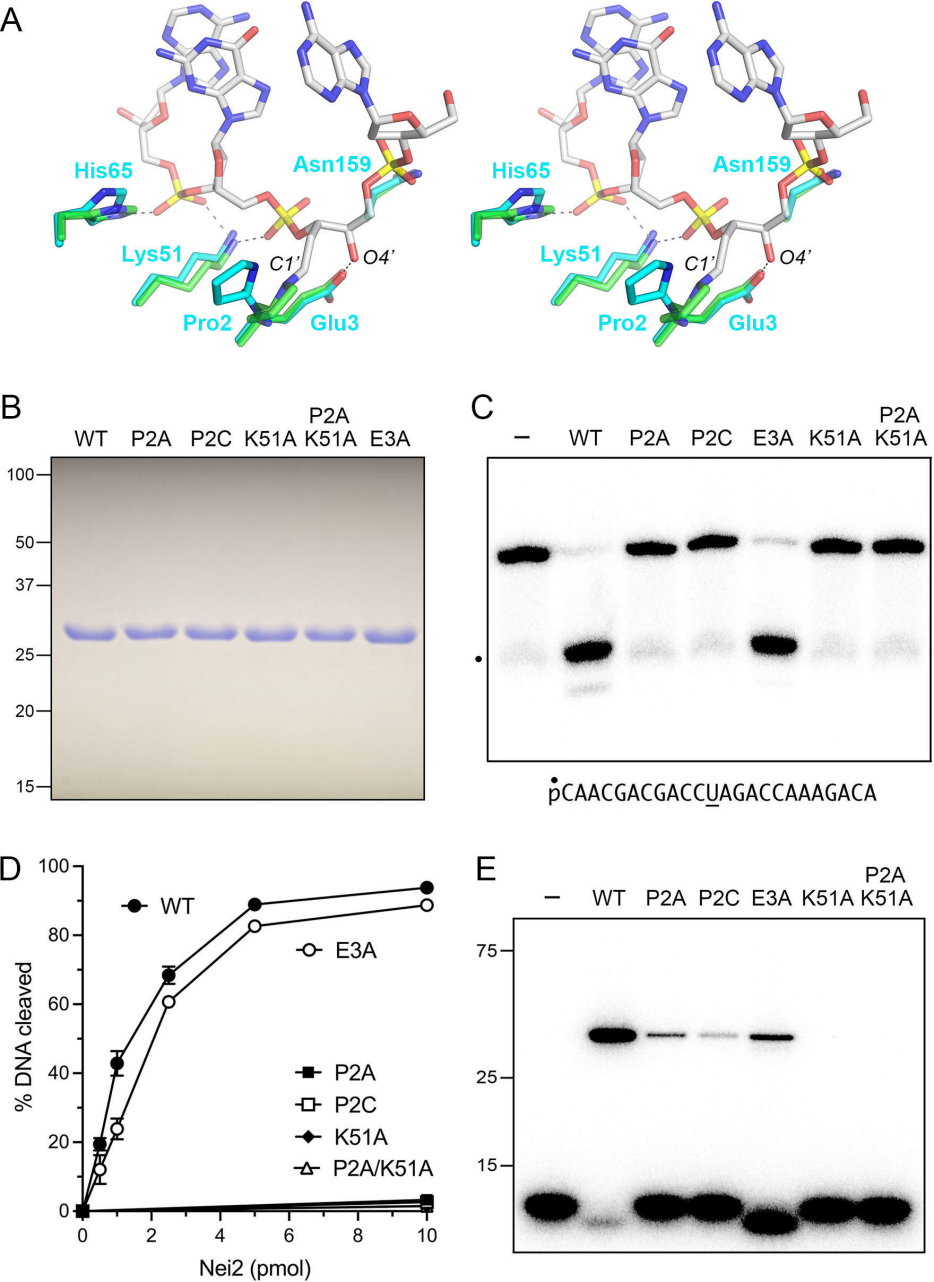
Nei2 active site and mutational effects on AP lyase activity. (**A**) The Nei2 structure was superimposed on the structure of the covalent EcoNei–DNA complex derived from the Schiff-base intermediate in the AP lyase reaction (PDB 1K3X). The figure shows a focused stereo view of the active site, highlighting covalent attachment of the EcoNei N-terminal proline to the C1′ atom of the ring-opened abasic deoxyribose and additional enzymatic contacts to the DNA in the vicinity of the AP site. EcoNei amino acids are rendered as stick models with green carbons: the DNA is a stick model with gray carbons. Equivalent Nei2 amino acids are depicted as stick models with cyan carbons. (**B**) Aliquots (10 µg) of purified recombinant wild-type Nei2 and the indicated mutants were analyzed by SDS-PAGE. The Coomassie blue-stained gel is shown. The positions and sizes (kDa) of marker polypeptides are indicated on the left. (**C**) AP lyase reaction mixtures (10 µL) containing 20 mM Tris-HCl, pH 8.0, 1 mM EDTA, 1 mM DTT, 100 nM (1 pmol) 5′ ^32^P-labeled 24-mer dU-containing DNA oligonucleotide (depicted at bottom), and 0.1 pmol *E. coli* UDG (from New England Biolabs) were pre-incubated at 25°C for 10 min. The reaction mixtures were supplemented with 10 pmol Nei2 and incubated at 25°C for 15 min. Reactions were quenched by addition of 10 µL of a solution containing 90% formamide (vol/vol), 50 mM EDTA, 0.1% (wt/vol) bromophenol blue, and 0.1% xylene cyanol. The reaction products were analyzed by electrophoresis through a 20% polyacrylamide gel containing 7.5 M urea in 89 mM Tris-borate, 2 mM EDTA. The radiolabeled DNAs were visualized by scanning the gel with a Typhoon FLA7000 imager. (**D**) AP lyase reactions were constituted as in panel (C) and contained varying amounts of Nei2 protein. The extents of DNA cleavage (incised DNA/total DNA in each lane) were calculated in ImageQuant and are plotted as a function of input Nei2. Each datum is the average of three separate Nei2 titration experiments ± SEM. (**E**) Borohydride trapping of covalent Nei2–(AP DNA) adduct. AP lyase reactions were constituted as in panel (C) except that 50 mM NaBH_4_ was included in the reaction mixtures prior to addition of Nei2. The reactions were quenched after 15 min incubation at 25°C by adding SDS sample buffer. The products were resolved by SDS-PAGE and visualized by scanning the gel. The positions and sizes (kDa) of marker polypeptides are indicated on the left.

A DALI search (20) of the protein database retrieved *E. coli* endonuclease VIII (EcoNei) as the closest structural homolog of *M. smegmatis* Nei2 (*Z* score 26.1; RMSD of 2.2 Å at 240 Cα positions). A structure-based alignment of the Nei2 and EcoNei primary structures highlighted 101 positions of amino acid side chain identity/similarity that includes the C-terminal tetracysteine Zn-binding motif (Fig. 4C). The structure of EcoNei has been solved at high-resolution as the covalent enzyme-DNA complex derived from the Schiff base intermediate in the AP lyase reaction (5). Figure 5A shows a superposition of the Nei2 and EcoNei–DNA structures, focusing in on the active site and enzymatic contacts to DNA at and near the AP site. Nei2 Pro2 corresponds to the EcoNei proline that engages in covalent adduct formation between its N atom and the C1′ atom of the deoxyribose at the abasic site. Nei2 Glu3 superimposes on an EcoNei glutamate that coordinates the O4′ atom of the ring-opened deoxyribose. Nei2 Lys51 superimposes on the equivalent lysine in EcoNei that coordinates the 3′-phosphate of the abasic nucleoside and the 3′-phosphate of the neighboring downstream nucleotide (Fig. 5A).

### Structure-guided mutagenesis identifies Nei2 amino acids essential for AP lyase activity

Based on the active site alignment to EcoNei, we conducted a mutational analysis of Nei2 amino acids Pro2, Glu3, and Lys51, replacing each singly with alanine. The P2A, E3A, and K51A proteins, and a P2A/K51A double mutant, were produced in *E. coli* with C-terminal His_6_-tags and purified in parallel with wild-type Nei2 (Fig. 5B). To test for AP lyase activity, we exploited a 24-mer 5′ ^32^P-labeled DNA oligonucleotide containing a single centrally placed uracil deoxynucleoside that was pretreated with *E. coli* uracil DNA glycosylase (UDG) to generate an abasic site. Reaction of 1 pmol of abasic DNA with 10 pmol of wild-type Nei2 at 25°C for 15 min resulted in efficient DNA incision at the AP site (Fig. 5C). The P2A and K51A mutants, and the P2A/K51A double-mutant, were inactive, while the E3A mutant retained AP lyase activity (Fig. 5C). Note that the very low level of cleaved DNA fragment seen in the absence of Nei2 (denoted by the dot next to lane—in Fig. 5C) was generated during the pre-treatment of the radiochemically pure 24-mer DNA substrate with *E. coli* UDG to generate the abasic site at which Nei2 acts. We also replaced Pro2 with cysteine (Fig. 5B), in light of the report that an N-terminal cysteine of *E. coli* lyase YedK can react with an AP site to form a Schiff base and then either: (i) proceed with β-elimination leading to release of incised DNA or (ii) form a stable covalent thiazolidine linkage at the ring-opened AP site (21). We found that the Nei2 P2C mutant was inactive as an AP lyase (Fig. 5C). Quantification of the extents of cleavage by 10 pmol of recombinant Nei2 protein (after correction for the no enzyme background) was as follows: WT, 93.8 ± 0.8%; E3A, 88.7 ± 1.4%; P2A, 3.3 ± 0.3%; P2C, 2.6 ± 0.15%; K51A, 1.5 ± 0.8%; and P2A/K51A, 1.5 ± 0.8% (Fig. 5D), whereby these values are the average of three independent experiments (±SEM). Titration of the wild type and E3A Nei2 preparations for AP lyase activity showed that product formation depended on input enzyme in both cases and that the specific activity of the E3A mutant was 80% of the wild-type enzyme (Fig. 5D).

Reaction of the UDG-pretreated abasic DNA (1 pmol) with 10 pmol wild-type Nei2 for 15 min in the presence of 50 mM NaBH_4_ resulted in the trapping of a covalent Nei2-DNA adduct that was detectable by SDS-PAGE (Fig. 5E). An additional product, migrating during SDS-PAGE slightly ahead of the input 24-mer DNA, corresponds to DNA cleaved by Nei2 that eluded trapping by borohydride at the Schiff base intermediate step. The reaction with wild-type enzyme resulted in covalent attachment of 73 ± 2.7% of the input DNA to the Nei2 protein. Parallel reactions with lyase-defective mutants P2A and P2C led to transfer of 6.1 ± 1.1% and 1.7 ± 1.1 % of the input DNA to the mutant enzymes, respectively. The reactions with lyase-defective mutants K51A and P2A/K51A yielded no detectable covalent DNA–Nei2 complex (Fig. 5E). The reaction with the lyase-competent E3A mutant generated only 11 ± 2.0% covalent DNA–Nei2 complex in the presence of borohydride while converting most of the input DNA into the incised lyase reaction product migrating ahead of the input 24-mer substrate. The extents of covalent DNA–Nei2 trapping by borohydride are averages of three independent experiments (±SEM).

### AP lyase activity is not necessary for Nei2 function in psoralen–UVA damage repair *in vivo*

**Fig 6:**
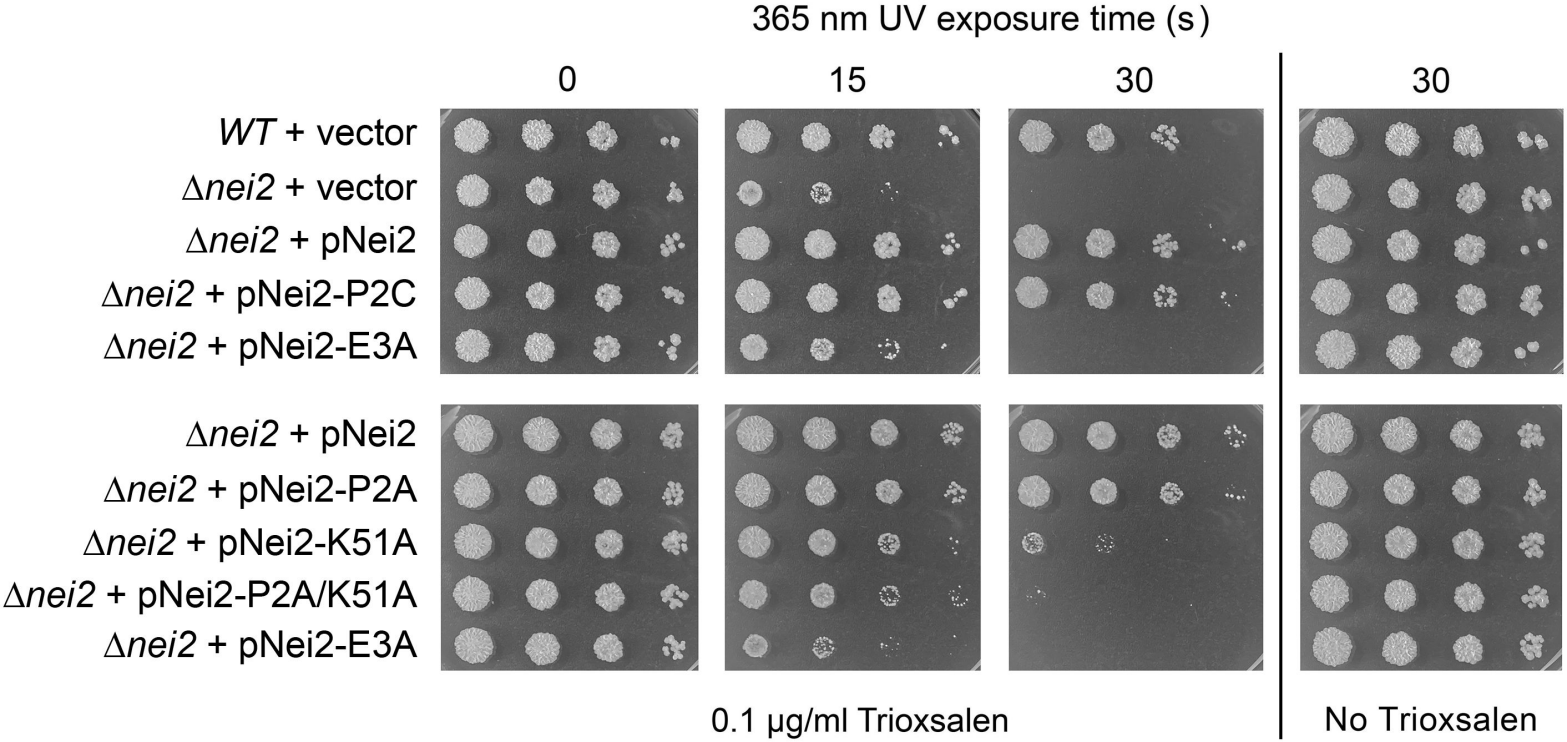
AP lyase activity is not required for Nei2 function in psoralen–UVA resistance. Serial 10-fold dilutions of wild-type and ∆*nei2* cells transformed with plasmids pMV261 (empty vector), pNei2-WT, pNei2-P2A, pNei2-P2C, pNei2-E3A, pNei2-K51A, or pNei2-P2A/K51A, were spotted on 7H10 agar plates lacking or containing 0.1 µg/mL trioxsalen and exposed to 365 nm light for 0, 15, or 30 s. The plates were photographed after incubation for 3 days at 37°C.

The set of biochemically characterized Nei2 mutants described above that either do or do not have AP lyase activity affords an opportunity to gauge whether Nei2 AP lyase is relevant to Nei2 function in resistance to trioxsalen–UVA damage. The mutant *nei2* alleles were placed on mycobacterial plasmids under the control of the *lhr–nei2* operon promoter and then tested in parallel with plasmid-borne wild-type *nei2* for complementation of the ∆*nei2* strain (Fig. 6). We were surprised to find that: (i) expression of AP lyase-defective Nei2 mutants P2A and P2C was just as effective as expression of wild-type Nei2 in rescuing the trioxsalen–UVA sensitivity of ∆*nei2* cells; (ii) expression of the AP lyase-active Nei2 mutant E3A was unable to complement the ∆*nei2* repair defect, that is, ∆*nei2* pNei2-E3A cells were just as sensitive to killing as ∆*nei2* cells bearing the empty vector (Fig. 6). Thus, AP lyase activity is neither sufficient nor necessary for Nei2’s function in evading trioxsalen–UVA damage *in vivo*. It was noteworthy that the Nei2 K51A mutant partially restored resistance to a 15 s and 30 s UVA exposure vis-à-vis the empty plasmid control (Fig. 6). Insofar as Nei2 Lys51 is predicted from the comparison to EcoNei to contribute to binding the DNA phosphodiesters, albeit not directly in catalysis at the AP site, we can speculate that the ability of Nei2 to interact with DNA is likely to be relevant to its repair function *in vivo*. Combining P2A with K51A did not affect the partial complementation of ∆*nei2* at 15 s UVA exposure but did attenuate rescue after 30 s exposure compared to the K51A single-mutant.

### Nei2 has 5-hydroxyuracil glycosylase activity

**Fig 7:**
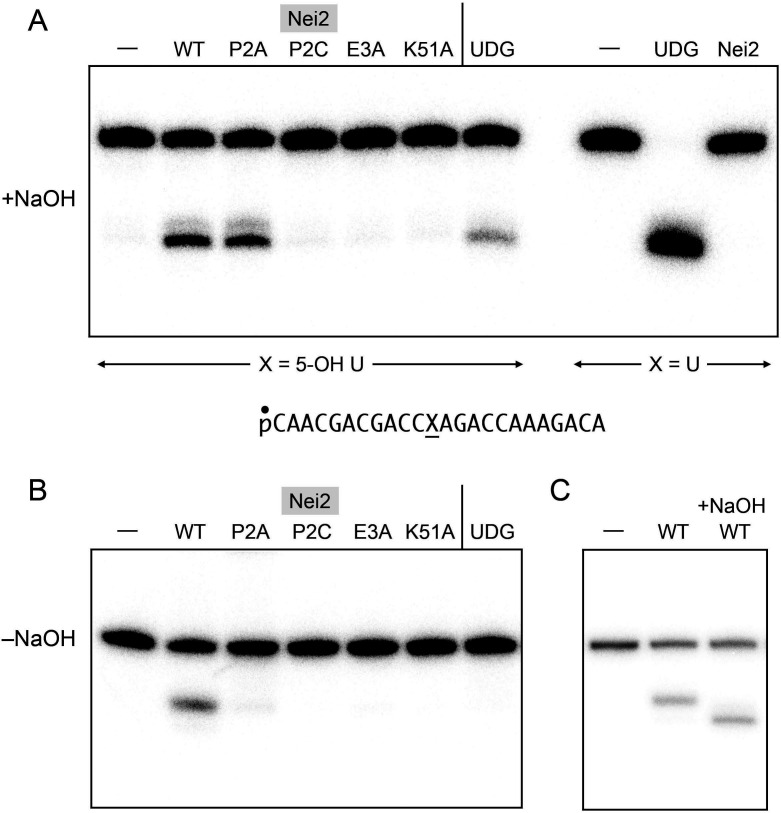
Nei2 has 5-hydroxyuracil glycosylase activity. (**A**) Glycosylase reaction mixtures (10 µL) containing 20 mM Tris-HCl, pH 8.0, 1 mM EDTA, 1 mM DTT, 100 nM (1 pmol) 5′ ^32^P-labeled 24-mer dX-containing DNA oligonucleotide (depicted at bottom, where X is 5-hydroxyuracil or uracil), and 10 pmol Nei2 or UDG as specified were incubated at 25 °C for 30 min. The reaction mixtures were adjusted to 0.2 M NaOH and heated for 5 min at 70°C. (**B**) Glycosylase reactions containing OH-U DNA and Nei2 or UDG as indicated were performed as described in panel (A), except that the NaOH treatment was omitted. (**C**) Reaction mixtures containing OH-U DNA and wild-type Nei2 (or a no enzyme control in lane –) were incubated at 25°C for 30 min and, where indicated by +NaOH, treated with 0.2 M NaOH and then neutralized by adding HCl to 0.2 M concentration. The other two samples were adjusted to 0.2 M NaCl. The reactions in panels (A–C) were quenched by addition of 10 µL of 90% formamide, 50 mM EDTA. The reaction products were analyzed by electrophoresis through a 20% polyacrylamide gel containing 7.5 M urea in 89 mM Tris-borate, 2 mM EDTA. The radiolabeled DNAs were visualized by scanning the gel with a Typhoon FLA7000 imager.

In our initial studies of Nei2, we did not detect a uracil DNA glycosylase activity remotely commensurate with its AP lyase activity (4). Given the genetic evidence that Nei2 function *in vivo* is directed toward at least one specific DNA lesion, a psoralen crosslink to the 5 and 6 carbons of thymine, it seemed plausible that the putative Nei2 glycosylase might have a fastidious substrate specificity for 5-substituted or 6-substituted pyrimidines. To test this speculation, we employed a 5′ ^32^P-labeled 24-mer oligonucleotide substrate with a centrally placed 5-hydroxyuracil (OH-U) nucleobase (Fig. 7A). We incubated 1 pmol of OH-U DNA with 10 pmol of wild-type Nei2 at 25°C for 30 min. The reaction was quenched with NaOH to promote β,δ-elimination of any abasic DNA that was generated by a Nei2 glycosylase activity. This procedure allows for the detection of glycosylase activity independent of lyase activity. PAGE analysis of the reaction mixture revealed that 32.4 ± 0.44% of the input OH-U DNA was converted by wild-type Nei2 into an alkali-cleavable product (Fig. 7A). Whereas the P2A mutant was as active as wild-type Nei2 as a glycosylase on the OH-U substrate (32.5 ± 0.2% product formation), the P2C, E3A, and K51A mutants were inactive (0.5%, 0.1%, and 0.1% product formation, respectively) (Fig. 7A). Of note, Nei2 was more active on the OH-U substrate than *E. coli* uracil DNA glycosylase (UDG), which converted 12.3 ± 0.5% of the input DNA into an alkali-cleavable abasic product (Fig. 7A). The extents of OH-U DNA cleavage cited are averages of three independent experiments (±SEM). Parallel glycosylase assays using an otherwise identical 24-mer DNA containing a single uracil nucleobase highlighted that *E. coli* UDG converted all of the input U DNA into an abasic product, whereas Nei2 was inactive as a uracil glycosylase (Fig. 7A). *E. coli* UDG is known to have more vigorous glycosylase activity in excising uracil versus 5-hydroxyuracil (22).

When the NaOH treatment was omitted, such that incision of the substrate depends on sequential glycosylase and lyase activities, the reaction with wild-type Nei2 resulted in conversion of 31 ± 0.8 % of the input OH-U substrate to a single product (Fig. 7B) that migrated with the species generated via β-elimination (Fig. 7C). As expected, the P2C, E3A, and K51A mutants, which did not form a product that was cleavable by alkali, failed to form a cleaved DNA when NaOH treatment was omitted (Fig. 7B). *E. coli* UDG, which has no lyase activity, also did not incise the OH-U DNA in the absence of alkali treatment. The salient finding here was that the P2A mutant incised only 2.7 ± 0.6% of the input OH-U DNA when alkali-treatment was withheld (Fig. 7B), suggesting that the P2A enzyme might be defective in converting the covalent Nei2–(abasic DNA) intermediate formed after ejection of the OH-U base into a β-elimination cleavage product.

**Fig 8:**
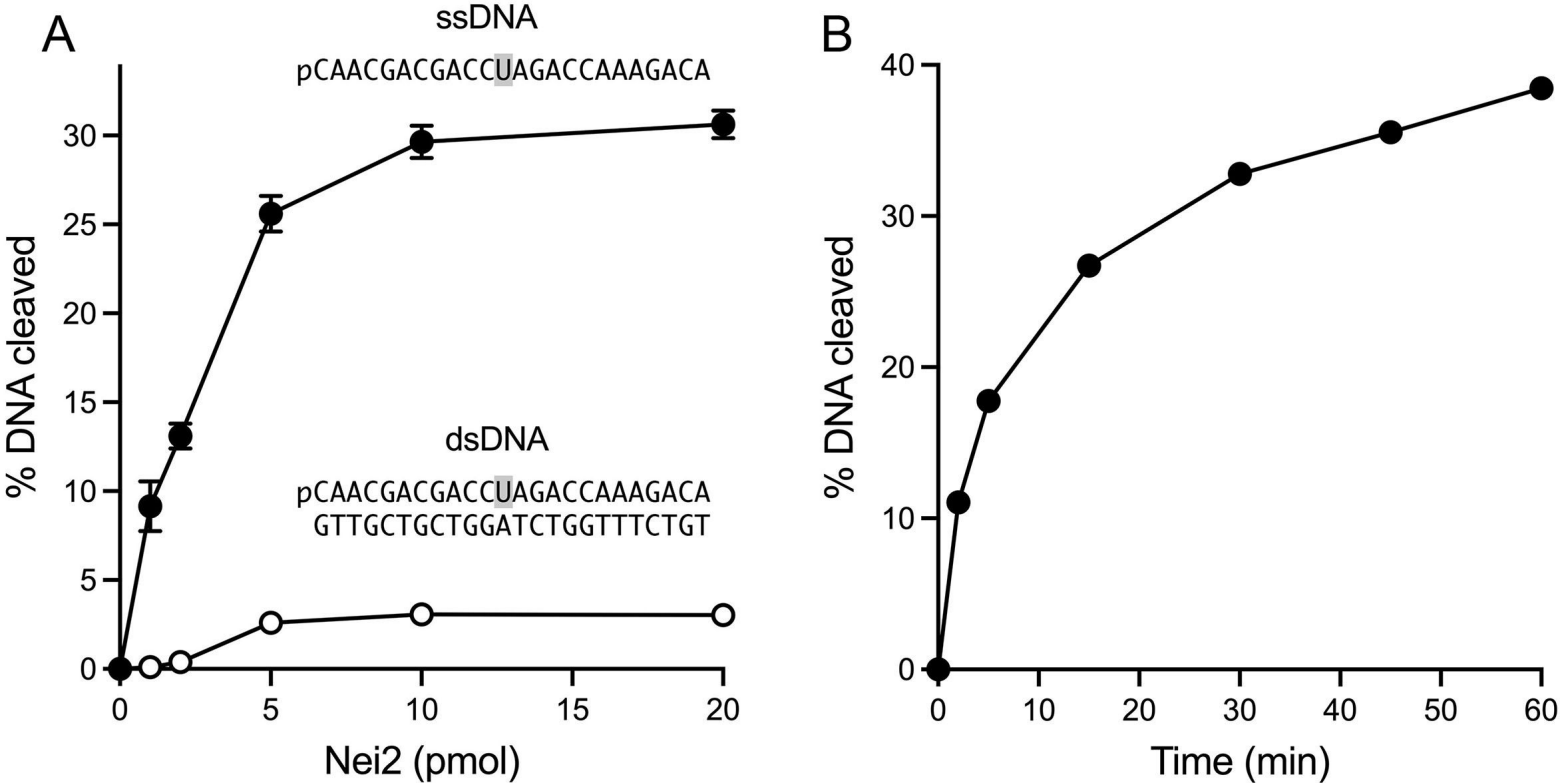
Nei2 5-hydroxyuracil glycosylase activity on single-strand versus double-strand DNA. (**A**) Reaction mixtures (10 µL) containing 20 mM Tris-HCl, pH 8.0, 1 mM EDTA, 1 mM DTT, 100 nM (1 pmol) of 5′-^32^P-labeled 24-mer single-strand (ss) DNA or 24 bp double-strand (ds) DNA with a single OH-U nucleobase in the labeled strand (shaded gray) and increasing Nei2 as specified on the *x*-axis were incubated at 25°C for 30 min. The reactions were quenched with formamide/EDTA, and the products were resolved by urea-PAGE. The extents of ssDNA or dsDNA cleavage are plotted as a function of input Nei2. Each datum is the average of three independent titration experiments ± SEM. (**B**) Reaction mixtures containing 20 mM Tris-HCl, pH 8.0, 1 mM EDTA, 1 mM DTT, 100 nM 5′-^32^P-labeled 24-mer OH-U ssDNA, and 2 µM Nei2 were incubated at 25°C. The reactions were quenched at times specified on the *x*-axis by adding an equal volume of formamide/EDTA. The extent of ssDNA cleavage is plotted as a function of incubation time. Each datum is the average of three independent time-course experiments ± SEM. The error values fall within the data symbols and are therefore not visible in the graph.

Titrations of wild-type Nei2 for glycosylase/β-lyase activity with the 24-mer single-stranded OH-U DNA substrate (1 pmol) showed that strand cleavage increased with Nei2 up to 5 pmol and plateaued at 10–20 pmol with 31% of the substrate incised during a 30 min incubation (Fig. 8A). The incised product formed by 10 pmol of Nei2 accumulated steadily with incubation time up to 15 min and then increased at a slower rate between 15 and 60 min, at which point 38% of the OH-U DNA was incised (Fig. 8B). To gauge whether Nei2 was active as a hydroxyuracil glycosylase on duplex DNA, we annealed the 24-mer 5′ ^32^P-labeled OH-U DNA oligonucleotide to a complementary unlabeled 24-mer DNA and isolated the blunt-end 24 bp duplex by preparative native gel electrophoresis. (Analytical native PAGE affirmed that the radiolabeled strand was in a duplex form that migrated more slowly than the labeled single strand.) Parallel titrations employing the 24 bp duplex OH-U DNA substrate highlighted that the extent of cleavage of the duplex substrate at saturating Nei2 was 10-fold lower than that of the single-stranded DNA (Fig. 8A).

We proceeded to show that Nei2 was active as a glycosylase with 24-mer single-stranded DNA substrates containing a single thymine glycol (Tg) or dihydrothymine (dhT) nucleobase (Fig. S4). The extents of excision of Tg (6.5 ± 0.66% of DNA cleaved) and dhT (4.5 ± 0.48% of DNA cleaved) by 10 pmol Nei2 (Fig. S4) were lower than what was observed for OH-U (Fig. 7).

### Features of Lhr needed for its role in psoralen–UVA damage repair *in vivo*

**Fig 9:**
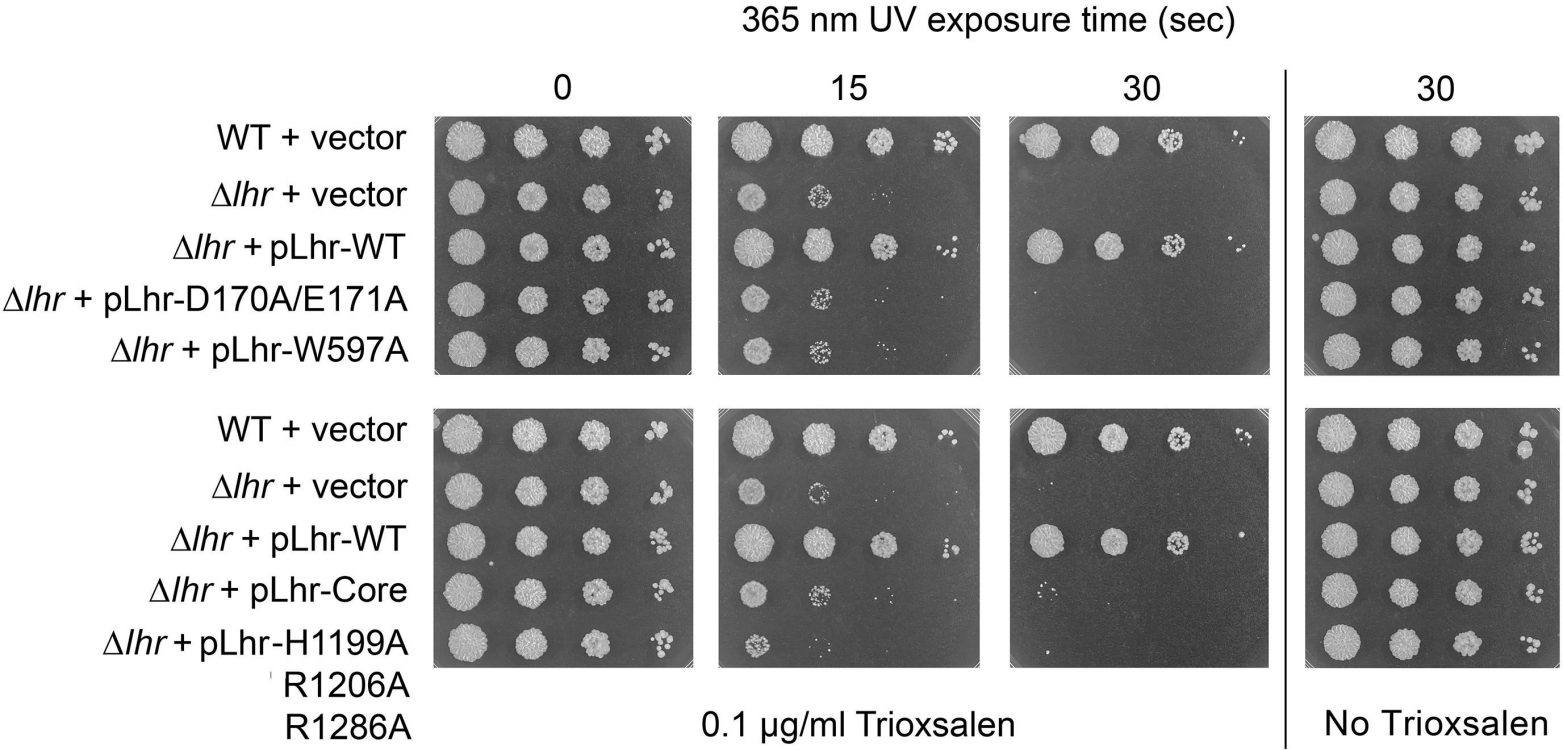
ATPase, helicase, and tetramerization are required for Nei2 function in psoralen–UVA resistance. Serial 10-fold dilutions of wild-type and ∆*lhr* cells transformed with plasmids pMV261 (vector), pLhr-WT, pLhr-D170A/E171A (ATPase-defective), pLhr-W597A (ATPase active, helicase-defective), pLhr-Core, or pLhr-H1199A-R1206A-R1286A (tetramerization-defective) were spotted on 7H10 agar plates lacking or containing 0.1 µg/mL trioxsalen and exposed to 365 nm light for 0, 15, or 30 s. The plates were photographed after incubation for 3 days at 37°C.

Previously, we conducted a series of ∆*lhr* complementation assays with biochemically defined Lhr mutants to gauge which activities and structural properties of Lhr are pertinent to its activity in MMC and cisplatin resistance *in vivo* (4). The mutant *lhr* alleles tested for complementation were *D170A/D171A* (ATPase-defective), *W597A* (ATPase active, helicase defective), *lhr-(1-856*) (Core helicase domain, lacking the CTD), and *H1199A/R1206A/R1286A* (tetramerization-defective). None of these mutant alleles restored MMC/cisplatin-resistance to ∆*lhr* cells (4). Here, we find that the same set of *lhr* alleles was unable to complement the trioxsalen–UVA sensitivity of ∆*lhr* cells (Fig. 9). We concluded that Lhr’s crosslink repair activities *in vivo* are contingent on the CTD and its ability to nucleate a homo-tetrameric Lhr quaternary structure and dependent on both ATP hydrolysis and duplex unwinding—independent of the type of DNA crosslink involved.

## DISCUSSION

The present study enhances our understanding of the DNA repair repertoire of the mycobacterial helicase Lhr and its operonic neighbor Nei2. In brief, Lhr helicase activity is needed to protect *M. smegmatis* from lethality inflicted by three different clastogens—MMC, cisplatin, and psoralen–UVA—that generate three different types of DNA inter-strand crosslinks. By contrast, the Nei2 protein (but not its AP lyase activity) is required to evade killing by psoralen–UVA exclusively. We suggest that Lhr anchors a bacterial pathway of DNA crosslink repair, populated by additional enzymes and proteins that direct and assist Lhr in the correction of particular types of crosslinked lesions. Nei2 is clearly one such factor, insofar as it plays no apparent role in Lhr-driven avoidance of killing by MMC and cisplatin.

Two features of Nei2 physiology stand out: (i) its specificity in fending off psoralen–UVA killing; and (ii) the uncoupling of Nei2 biological activity in psoralen–UVA survival from its biochemical activity as an AP lyase. Two possible explanations for why the ∆*nei2* mutant is not sensitized to MMC and cisplatin are that: (i) Nei2 is functionally redundant to other mycobacterial proteins in an MMC/cisplatin sub-pathway of Lhr-driven crosslink repair; or (ii) Nei2 cannot facilitate repair of MMC/cisplatin crosslinks and this function is executed by other MMC/cisplatin-specific repair factors. The potential candidates for such factors include the three other members of the Nei/Fpg-family of predicted glycosylase/lyase enzymes in the *M. smegmatis* proteome: Nei1, Fpg1, and Fpg2. Prior studies showed that ∆*nei1*, ∆*nei2*, ∆*fpg1*, and ∆*fpg2* single-deletions had no effect on *M. smegmatis* growth under laboratory conditions, nor did ∆*nei1* ∆*nei2* and ∆*fpg1* ∆*fpg2* double-deletions (23). The ∆*nei1* ∆*nei2* and ∆*fpg1* ∆*fpg2* double-deletions had no effect on *M. smegmatis* sensitivity to killing by hydrogen peroxide or UVC (23). In light of the present results anent Nei2, it will be of interest in future studies to interrogate genetically whether Nei1, Fpg1, and Fpg2 contribute to DNA crosslink repair and whether their contributions, if any, are contingent on their enzymatic activities.

We reported here the atomic structure of *M. smegmatis* Nei2 and, based on alignment to the structure of EcoNei in its trapped complex with DNA (5), conducted a mutational analysis that revealed the essentiality of Pro2 and Lys51 (but not Glu3) for Nei2 AP lyase activity *in vitro*. The requirement for an N-terminal proline as the site of DNA Schiff base formation is characteristic of Nei/Fpg-type glycosylase/lyase enzymes. However, mammalian NEIL3 is an exception in that it has a valine at this position and production of active NEIL3 requires the removal of the encoded Met1 amino acid (24, 25). The presence of a glutamate adjacent to the active site proline (or valine in NEIL3) is a conserved feature of Nei/Fpg-family enzymes. In the case of EcoNei and EcoFpg, it was shown that mutating the glutamate exerts a strong negative effect on glycosylase activity yet is comparatively benign as regards AP lyase activity (26, 27). This is consistent with the observation here that the recombinant Nei2 E3A mutant retains substantial AP lyase activity.

We demonstrate that *M. smegmatis* Nei2 has 5-hydroxyuracil DNA glycosylase activity *in vitro* and that the active site constituents required for its glycosylase activity are distinct from those needed for its AP lyase activity. Most conspicuously, the 5-hydroxyuracil glycosylase is abolished by the E3A mutation that spares the AP lyase, while the P2A mutation that is severely deleterious to AP lyase is benign with respect to 5-hydroxyuracil glycosylase. Glu3, which makes a hydrogen bond to the ring-opened deoxyribose 4′-OH of the borohydride-trapped covalent EcoNei–(abasic DNA) intermediate (5), is proposed to protonate the deoxyribose O4′ leaving group during the first step of the glycosylase reaction in which the proline nitrogen nucleophile attacks the C1′ atom to which the damaged base remains covalently bound (5, 28). Among the Nei2 alanine mutants, the E3A and K51A variants that are defective as 5-hydroxyuracil DNA glycosylases *in vitro* are unable to complement the psoralen–UVA sensitivity of the ∆*nei2* strain, whereas the P2A variant retains 5-hydroxyuracil DNA glycosylase activity *in vitro* and psoralen crosslink repair activity *in vivo*. These findings would suggest that the glycosylase activity of Nei2 (but not its stand-alone AP lyase activity, uncoupled from a glycosylase step) is pertinent to its DNA crosslink repair capacity. The fly in the ointment is the P2C mutant, which is active in complementing ∆*nei2* psoralen sensitivity but defective as a 5-hydroxyuracil glycosylase. It is conceivable that the P2C variant retains glycosylase activity against lesions other than 5-hydroxyuracil (e.g., psoralen-adducted thymine) and that this could account for its complementation activity. There is precedent in the case of human NEIL1 for a single amino acid difference resulting in selective loss of activity against some damaged bases while preserving activity against other lesions (29).

The fact that the tetrameric quaternary structure of Lhr helicase is required for its activity against three different inter-strand crosslinkers suggests that it might provide a scaffold for assembly of a crosslink repair complex, potentially with four independent docking sites for mycobacterial proteins (including Nei2) that help execute the repair process. Our attempts to demonstrate the formation of a binary Lhr·Nei2 complex *in vitro*, by mixing the recombinant proteins and ensuing gel filtration, have not been fruitful, which might suggest either that their interaction entails co-occupancy on a lesion-containing DNA or that their interaction depends on one or more additional mycobacterial protein(s).

We noted previously (4) certain biochemical similarities between Nei2 and eukaryal NEIL3, a glycosylase/lyase that catalyzes unhooking of inter-strand psoralen and abasic site crosslinks *in vivo* and *in vitro* (30–34). Like Nei2, NEIL3 prefers single-stranded substrates versus duplex DNA. NEIL3 is also active on fork structures in which an abasic site crosslink is situated at the duplex/single-strand junction (31). The analogy is fortified by the present evidence that Nei2, like NEIL3, acts to evade psoralen crosslinks *in vivo*. NEIL3 is recruited to crosslink sites by physical interaction with the ubiquitin-modified CMG helicase (32, 35). Recent studies suggest a noncatalytic role for NEIL3 in cisplatin crosslink repair, whereby it acts as a scaffold protein that recruits the proteasome to sites of DNA damage (36). Mycobacteria elaborate a Pup-proteasome system (analogous to the eukaryal Ub-proteasome system) that is genetically linked to the PafBC transcription factor that regulates the *lhr–nei2* operon in response to DNA damage (11, 13, 37). Whereas Lhr and Nei2 were not represented in the mycobacterial pupylome as initially mapped in unstressed cells (38, 39), it is likely that any potential regulation of DNA repair proteins by pupylation would be evident only in response to DNA damage. Indeed, the Weber-Ban lab has identified Nei2 as a pupylated protein in *M. smegmatis* cells exposed to MMC (11). To see if interdicting pupylation affects the sensitivity of *M. smegmatis* to DNA crosslinkers, we treated pupylation-competent wild-type and pupylation-defective ∆*dop* cells with trioxsalen–UVA, MMC, and cisplatin and observed no apparent survival defect in the absence of pupylation at crosslinker exposure levels that were lethal to ∆*lhr* cells (Fig. S5). This result might signify either: (i) that pupylation of Nei2 is of no functional consequence vis-à-vis crosslink repair; or (ii) that pupylation and proteosome targeting of Nei2 protein induced in response to DNA damage serves to restore basal levels of Nei2 after recovery from DNA damage stress, as has been demonstrated for mycobacterial RecA (11).

## MATERIALS AND METHODS

### Deletion of *nei2* in *M. smegmatis*

Deletion of the entire 252-aa open reading frame (ORF) of *nei2* in *M. smegmatis* was achieved by allelic exchange with a hygromycin-resistance cassette (*hygR*). Briefly, a DNA fragment consisting of the 500 bp upstream of the annotated *nei2* start codon was PCR-amplified from *M. smegmatis* mc^2^155 genomic DNA with primer GW01 (5′-GTCATCGTAAGCTTCTGGGCGGTGCCCAGTTCGC) and primer GW02 (5′-GTCGCATCTAGAGCGGCCGCCTACGCGGTGTGGAACACGGTG). A 3'-flanking genomic fragment starting from the *nei2* stop codon and spanning 500 bp of downstream DNA was PCR-amplified with primer GW03 (5′-GTCGTACTCGAGTGACTGCGGCGACCGGAAATAG) and primer GW04 (5′-GTCATCGTTCTAGAACCTACGTCGACCCGGCGGGCG). The PCR fragments were inserted upstream and downstream of a *hygR* cassette in pUC19. The resulting plasmid pGW01 was digested with HindIII and XbaI. The excised fragment containing the *nei2* 5′-flank, *hygR*, and the *nei2* 3′-flank was gel-purified and then transformed into *M. smegmatis* mc^2^155 strain MGM1957 (bearing *kanR*-marked recombineering plasmid pJV48) that had been induced with acetamide for 3 h at 37°C to express mycobacteriophage Che9c proteins Gp60 and Gp61 (40). After the selection of transformants for hygromycin resistance and then screening for passive loss of the pJV48 plasmid (manifest by sensitivity to kanamycin), candidate hygromycin-resistant, kanamycin-sensitive ∆*nei2* strains were genotyped by diagnostic PCR using primers flanking and internal to the *hygR* gene disruption cassette. The ∆*nei2* strains used in this study were confirmed by PCR-amplifying and sequencing the ∆*nei2::hygR* locus.

### Complementation of ∆*nei2* by expression of wild-type Nei2 and Nei2 mutants

A DNA fragment encoding Nei2 was PCR-amplified from *M. smegmatis* genomic DNA with primers that introduced a HindIII site downstream of the native stop codon. A second DNA fragment containing the *lhr* promoter region was PCR-amplified from *M. smegmatis* genomic DNA with primers that introduced a NotI site 225 bp upstream of the *lhr* start codon. The DNA fragments containing the *lhr* promoter region and *nei2* were fused in a two-stage overlap extension PCR step. The PCR product was digested with NotI and HindIII and inserted between the NotI and HindIII site of the mycobacterial plasmid vector pMV261 (marked with a kanamycin-resistance gene, *kanR*), thereby yielding pNei2. Nei2 missense mutations P2A, P2C, E3A, K51A, and P2A/K51A were introduced by PCR amplification of the *nei2* ORF with mutagenic primers. The mutant PCR products were digested with NotI and HindIII and cloned into pMV261. All plasmid inserts were sequenced to verify that no unintended coding changes were acquired during amplification and cloning. Wild-type and mutant pNei2 plasmids were transformed into the *M. smegmatis* ∆*nei2* strain. Wild-type and ∆*nei2* cells were transformed in parallel with the empty pMV261 vector.

### Sensitivity to trioxsalen–UVA

Log phase cultures (1.5 mL at *A*_600_ of 0.3–0.4) of *M. smegmatis* strains grown in Middlebrook 7H9 medium were diluted in 10-fold increments and aliquots (2.5 µL) were spotted 7H10 agar plates supplemented with 0.1 µg/mL trioxsalen (from Thermo Scientific; prepared as a 1 mg/mL stock solution in DMSO), 0.5% glycerol, and 0.5% dextrose. The agar plates were incubated at 37°C for 2 h to allow uptake of trioxsalen and then UVA-irradiated for the times specified with a 100 W 365 nm LED lamp (Everbeam) placed 10 cm above the plate. Immediately after exposure, the plates were wrapped in foil and incubated at 37°C for 3 days. Trioxsalen sensitivity experiments were performed with three independent biological replicates; representative experiments are shown in Fig. 1, 6 and 9.

### Sensitivity to mitomycin C

Log phase cultures (1.5 mL at *A*_600_ of 0.3–0.4) of *M. smegmatis* strains grown in Middlebrook 7H9 medium were supplemented with mitomycin C (from Sigma; prepared as a 0.8 mg/mL stock solution in DMSO) at the concentrations specified in figure legends. After incubation for 2 h at 37°C with constant shaking (200 rpm), the control and MMC-treated cells were harvested by centrifugation, washed two times with drug-free 7H9 medium, and resuspended in 7H9 medium to attain equal optical density. Aliquots (2.5 µL) of serial 10-fold dilutions were spotted on 7H10 agar plates supplemented with 0.5% glycerol, 0.5% dextrose, and incubated at 37°C for 3 days. MMC sensitivity experiments were performed with three independent biological replicates; a representative experiment is shown in Fig. 2.

### Sensitivity to cisplatin

Log phase cultures (1.5 mL at *A*_600_ of 0.3–0.4) of *M. smegmatis* strains grown in 7H9 medium were supplemented with cisplatin (from US Pharmacopeia, prepared as a 2 mg/mL stock solution in 150 mM NaCl) at the concentrations specified in figure legends. After incubation for 1 h at 37°C with constant shaking (200 rpm), the control and cisplatin-treated cells were harvested by centrifugation, washed once with drug-free 7H9 medium, and resuspended in 7H9 medium to attain equal optical density. Aliquots (2.5 µL) of serial 10-fold dilutions were spotted on 7H10 agar plates supplemented with 0.5% glycerol, 0.5% dextrose, and incubated at 37°C for 3 days. Cisplatin sensitivity experiments were performed with three independent biological replicates; a representative experiment is shown in Fig. 2.

### Sensitivity to UVC irradiation

*M. smegmatis* strains were grown to log phase (*A*_600_ 0.3–0.4) and serial 10-fold dilutions prepared in 7H9 media were spotted on 7H10 agar plates supplemented with 0.5% glycerol and 0.5% dextrose. UVC irradiation at the doses specified in the figures was performed with a Spectrolinker XL-1500 UV crosslinker (Spectronic Corp.) fitted with 254 nm bulbs. Immediately after exposure, the plates were wrapped in foil (to prevent repair by photolyase) and incubated at 37°C for 3 days. UVC sensitivity experiments were performed with three independent biological replicates; a representative experiment is shown in Fig. 3.

### Sensitivity to MMS

Log phase cultures (1.5 mL at *A*_600_ of 0.3–0.4) of *M. smegmatis* strains grown in 7H9 medium were supplemented with methyl methanesulfonate (from Sigma, stored as a 1.3 g/mL stock solution in 100% DMSO) at the concentrations specified in figure legends. After incubation for 1 h at 37°C with constant shaking (200 rpm), cold sodium thiosulfate was added to 5% (vol/vol) final concentration. The control and MMS-treated cells were harvested by centrifugation, washed once with drug-free 7H9 medium, and resuspended in 7H9 medium to attain equal optical density. Aliquots (2.5 µL) of serial 10-fold dilutions were spotted on 7H10 agar plates supplemented with 0.5% glycerol, 0.5% dextrose, and incubated at 37°C for 3 days. MMS sensitivity experiments were performed with two or three independent biological replicates; a representative experiment is shown in Fig. 3.

### Recombinant Nei2

The pET21b-Nei2 plasmid for bacterial expression of wild-type Nei2 fused to a C-terminal His_6_ tag was described previously (4). Missense mutations were introduced into the Nei2 ORF by two-stage overlap extension polymerase chain reaction using mutagenic primers. PCR fragments containing the mutated ORFs were inserted into plasmid pET21b. All plasmid inserts were sequenced to verify that no unintended coding changes were acquired during amplification and cloning. Recombinant Nei2 and Nei2 mutants were produced in *E. coli* BL21(DE3) and purified from soluble extracts of 1 L cultures by nickel-affinity and Superdex-200 gel filtration steps as described previously (4). Peak fractions were pooled, concentrated by centrifugal ultrafiltration, frozen, and stored at –80°C. Protein concentrations were determined with the BioRad dye reagent using BSA as the standard.

### Crystallization of Nei2 and structure determination

Crystals of Nei2 were grown by sitting drop vapor diffusion at 22°C. Aliquots (1 µL) of 87 µM Nei2 in 25 mM Tris-HCl, pH 8.0, 150 mM NaCl, and 0.5 mM TCEP were mixed with an equal volume of reservoir solution containing 0.2 M ammonium acetate, 0.1 M Bis-Tris pH 5.5, and 25% PEG-3350. Crystals were harvested and made into a seed stock using a seed bead kit (Hampton Research). Aliquots (1.5 µL) of 346 µM protein were mixed with 0.5 µL seed stock and 2 µL of reservoir solution containing 0.2 M ammonium acetate, 0.1 M Bis-Tris pH 5.5, and 15% PEG-3350. Crystals that grew after 2–4 days were cryoprotected in paraffin oil and then flash-frozen in liquid nitrogen. X-ray diffraction data from a single Nei2 crystal were collected at the Advanced Photon Source beamline 24ID-C. The crystal diffracted to 1.45 Å resolution, belonged to space group P2_1_2_1_2_1_, and contained one protomer in the ASU. Reduction of the crystallographic data were performed using XDS (41) and AIMLESS (42, 43). The Nei2 structure was determined by molecular replacement as implemented in PHENIX.PHASER (44), using an AlphaFold search model (alphafold.ebi.ac.uk/entry/A0QT90). The Nei2 structure was iteratively improved in PHENIX.REFINE interspersed with manual adjustments of the model in COOT (45). Data collection and refinement statistics are compiled in Table S1. The Nei2 structure has been deposited in the RCSB Protein Data Bank (PDB ID 8TJG).

## SUPPLEMENTAL MATERIAL

Supplemental Figures and Table mbio.01248-24-s0001.pdf Figures S1 to S5; Table S1.

ASM does not own the copyrights to Supplemental Material that may be linked to, or accessed through, an article. The authors have granted ASM a non-exclusive, world-wide license to publish the Supplemental Material files. Please contact the corresponding author directly for reuse.
