# Supplementary material for: Structure and in vivo psoralen DNA crosslink repair activity of mycobacterial Nei2
Source: mBio. 2024 Jul 16;15(8):e01248-24. doi: 10.1128/mbio.01248-24 (PMC11323726; doi:10.1128/mbio.01248-24)
Supplement: Supplemental Figures and Table — Figures S1 to S5; Table S1. [file mbio.01248-24-s0001.pdf]

Supporting Information for

Structure and in vivo psoralen DNA crosslink repair activity of mycobacterial Nei2

Garrett M. Warren and Stewart Shuman

Figures S1, S2, S3, S4, S5

Table S1

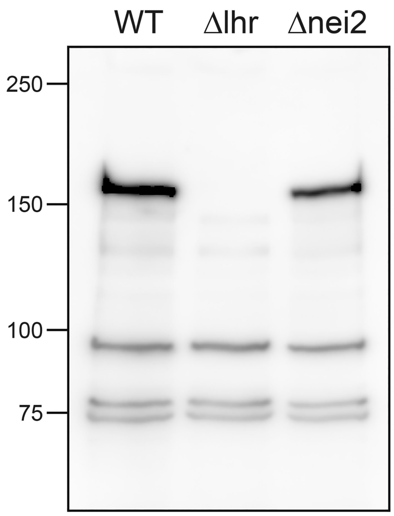

Figure S1. Lhr expression is unaffected by ablation of Nei2. Anti-Lhr Western blot of whole-cell extracts of wild-type, Δ*lhr*, and Δ*nei2* strains. The positions and sizes (kDa) of marker polypeptides are indicated on the left. The immunoreactive ~175 kDa Lhr polypeptide is absent in the Δ*lhr* strain. Smaller non-specific immunoreactive polypeptides present in all samples served as a loading control. Preparation and affinity purification of the antibody, bacterial extract preparation, and Western blotting were performed as described previously (4).

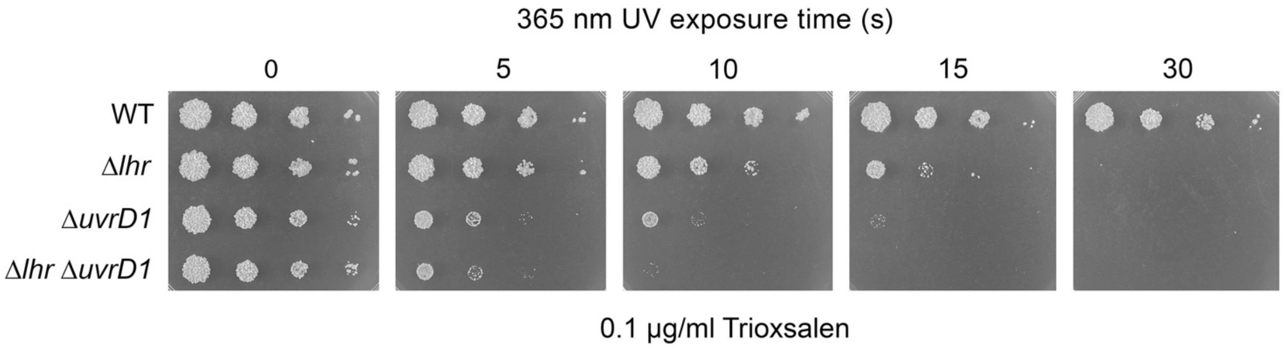

Figure S2. Contributions of Lhr and UvrD1 to repair of psoralen–UVA damage. Serial ten-fold dilutions of wild-type, Δ*lhr*, Δ*uvrD1*, and Δ*lhr* Δ*uvrD1* cells were spotted on 7H10 agar plates containing 0.1 µg/ml trioxsalen and exposed to 365 nm light for 0, 5, 10, 15, or 30 s. The plates were photographed after incubation for 3 d at 37˚C.

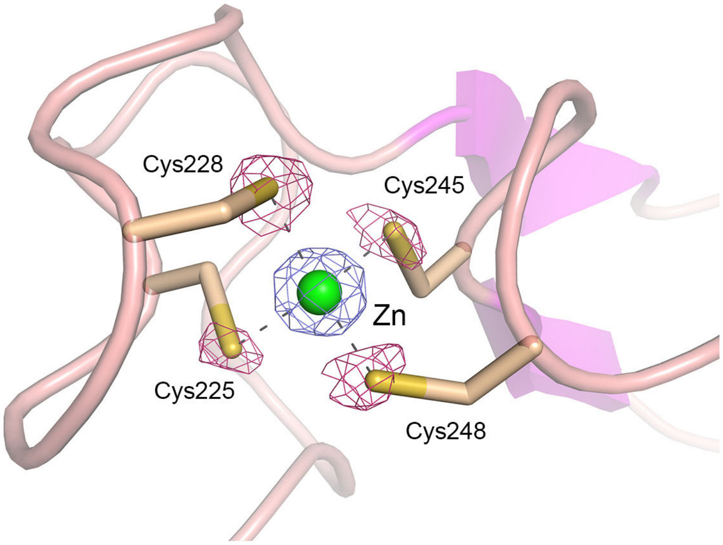

Figure S3. Zinc-binding site of Nei2. A single zinc atom (green sphere) is coordinated by four cysteine Sγ atoms as shown. Anomalous difference peaks overlying the Zn atoms (blue mesh) and the cysteine Sγ atoms (red mesh) are contoured at 16σ and 2.5σ, respectively.

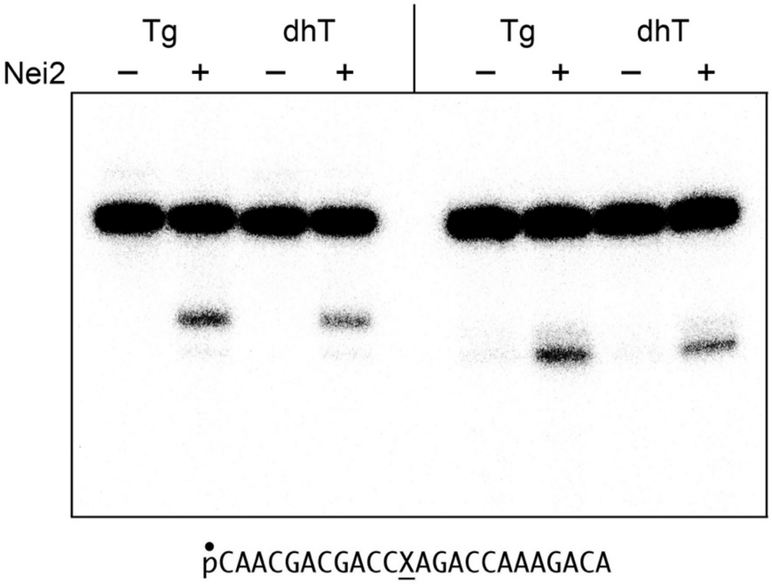

Figure S4. Nei2 has thymine glycol and dihydrothymine glycosylase activities. Glycosylase reaction mixtures (10 µl) containing 20 mM Tris-HCl, pH 8.0, 1 mM EDTA, 1 mM DTT, 100 nM (1 pmol) 5' ^{32}P-labeled 24-mer dX-containing DNA oligonucleotide (depicted at bottom, where X is thymine glycol Tg or dihydrothymine dhT), and 10 pmol Nei2 (where indicated by +) were incubated at 25˚C for 30 min. The reaction mixtures on the right were adjusted to 0.2 M NaOH and heated for 5 min at 70˚C. The NaOH treatment was omitted for the reactions on the left. The reaction products were analyzed by electrophoresis through a 20% polyacrylamide gel containing 7.5 M urea in 89 mM Tris-borate, 2 mM EDTA. The radiolabeled DNAs were visualized by scanning the gel with a Typhoon FLA7000 imager. The extents of excision of Tg (6.5 ± 0.66% of DNA cleaved) and dhT (4.5 ± 0.48% of DNA cleaved) cited are the averages of three independent experiments ±SEM.

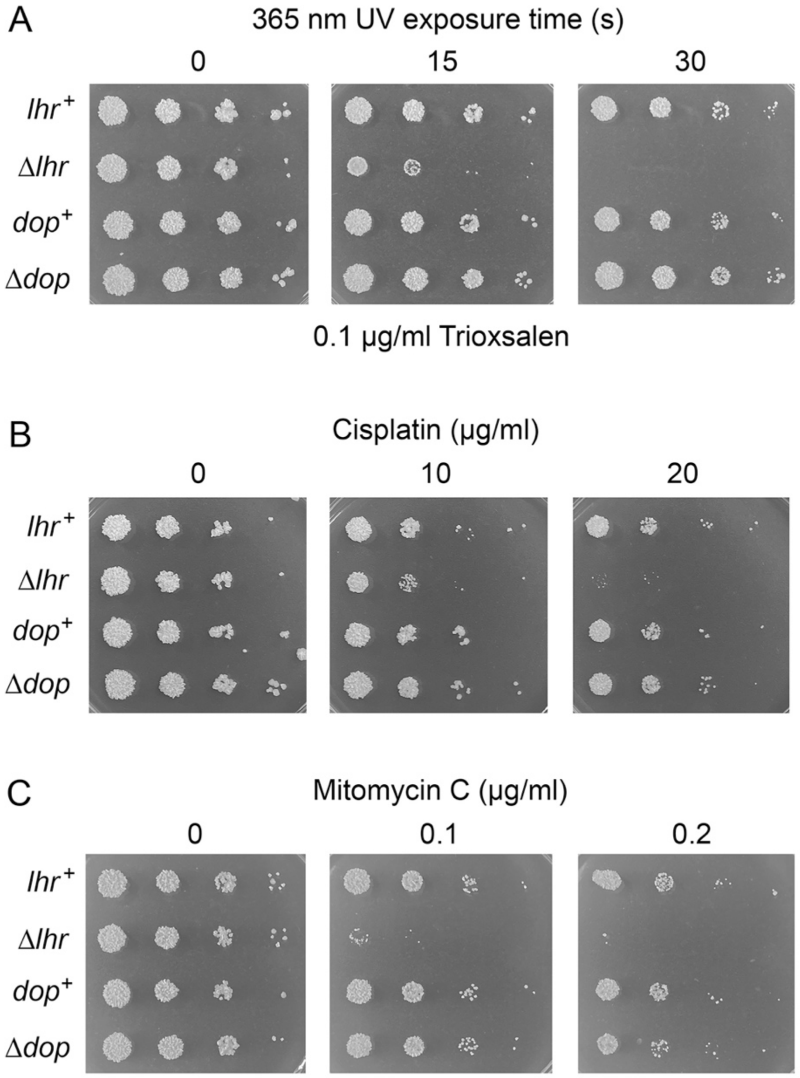

Figure S5. Deletion of *dop* does not sensitize *M. smegmatis* to DNA crosslinkers. (A) Serial ten-fold dilutions of *M. smegmatis* mc^{2}155 *lhr*$^+$ (wild-type) and Δ*lhr* cells and *M. smegmatis* SMR5 *dop*$^+$ (wild-type) and Δ*dop* cells were spotted on 7H10 agar plates containing 0.1 µg/ml trioxsalen and exposed to 365 nm light for 0, 15, or 30 s. The plates were photographed after incubation for 3 d at 37˚C. (B,C) *lhr*$^+$, Δ*lhr*, *dop*$^+$, and Δ*dop* cells were treated with either 0, 10, or 20 µg/ml cisplatin for 1 h at 37˚C (panel B) or 0, 0.1, or 0.2 µg/ml MMC for 2 h at 37˚C (panel C). Post-treatment, the cells were harvested by centrifugation, washed twice to remove the clastogen, resuspended, and adjusted to equal optical density. Serial tenfold dilutions were spotted on 7H10 agar plates and incubated for 3 d at 37˚C to gauge survival. The *dop*$^+$ and Δ*dop* strains were obtained from Prof. Eilika Weber-Ban (ETH Zurich).

Table S1. Nei2 crystallographic data and refinement statistics

**Data collection**	
Beamline	APS 24-ID-C
Wavelength (Å)	0.9791
Space group	$P2_12_12_1$
Cell dimensions	
a, *b*, *c* (Å)	54.065, 57.177, 66.287
α, β, γ (°)	90, 90, 90
Resolution	66.29-1.45 (1.45-1.48)
No. of unique reflections	36563
$\langle I\rangle/\langle I\sigma\rangle$	19.6 (2.2)
R_{pim}	0.020 (0.305)
CC(1/2)	0.999 (0.782)
Redundancy	11.5 (5.7)
Completeness (%)	98.5 (83.8)
Wilson B-factor (Å)	16.45
**Refinement**	
Resolution (Å)	43.30-1.45
R_{work}/R_{free}	0.1669/0.2052
Rmsd from ideal geometry	
bond lengths (Å)	0.009
bond angles (°)	1.067
Ramachandran plot	
favored (%)	97.58
allowed (%)	2.02
outliers (%)	0.40
Average *B*-factors (Å^2)	17.3
**Model contents**	
Protomers/ASU	1
Protein residues	250
Ligands	Zn^{2+}
Water	245
PDB ID	8TJG

Data for highest resolution shell are shown in parenthesis.
R_{free} was computed for ~5% of randomly selected reflections.
